# Letter to the editor concerning the article ‘Association between rotavirus vaccination and risk of intussusception among neonates and infants: a systematic review and meta-analysis’ (JAMA Netw Open. 2019;2(10):e1912458)

**DOI:** 10.1080/21645515.2020.1730119

**Published:** 2020-03-18

**Authors:** Bernd Benninghoff, Priya Pereira, Corinne Willame

**Affiliations:** GSK Vaccines, Wavre, Belgium

**Keywords:** Rotavirus vaccines, intussusception, safety

## Abstract

A recent meta-analysis investigating the association between intussusception (IS) and rotavirus (RV) vaccination demonstrated an absence of risk up to 2 years after vaccination. Meta-analyses including only randomized clinical trials are inadequate to identify a potential increased risk of rare adverse events such as IS. The study conducted failed to discuss relevant limitations. Additionally, the safety profiles of newer RV vaccines, evaluated in clinical studies with limited sample size, were considered comparable with that of the well-established and widely used RV vaccines, RotaTeq and Rotarix. We, therefore, re-emphasize that extensive and updated evidence from post-marketing surveillance indicates a slight increased risk of IS, mostly within 7 days of RV vaccination, with a benefit/risk profile assessment in favor of RV vaccination.

A recent systematic review and meta-analysis by Lu et al.^1^ investigated the association between rotavirus (RV) vaccination and the risk of intussusception (IS). The review included phase 1 to 3 placebo-controlled studies on 5 RV vaccines, of which 2 (*Rotarix* and *RotaTeq*) are World Health Organization (WHO)-prequalified and widely used, with well-established immunogenicity, safety, and effectiveness against RV disease. The 3 other RV vaccines included in the meta-analysis are newer vaccines (2 WHO-prequalified, available locally, i.e., *Rotavac, Rotasiil*, and a human neonatal RV vaccine RV3BB, still in clinical development). The meta-analysis demonstrated no increased risk of IS up to 2 years after vaccination for all 5 vaccines.^1^

In the following, we highlight several noteworthy concerns about this work.

## 1. A Meta-analysis including only randomized clinical trials (RCTs) to assess rare safety events for RV vaccines is erroneous

While common adverse events are identified during clinical development phases, rare adverse events such as IS in case of RV vaccination may go undetected or unconfirmed until post-marketing use. Post-marketing evaluation and passive surveillance are needed to detect rare adverse events or association with diseases that have low incidences. Such safety evaluations have been previously requested by national health authorities as they provide strong evidence on the benefits and risks of real-world use of RV vaccines.

## 2. The conclusion statement “our results contradict the post-marketing monitoring suggestion about the risk of IS after RV vaccination” is incorrect

The scientific community and regulatory bodies largely acknowledge the increased risk of IS within a short interval after RV vaccination based on scientific methodological assessments.^2-4^ The increased risk of IS has been demonstrated in several large population-based studies.^5,6^ The authors missed to mention important limitations of their work, discussed below.

## 3. The review fails to highlight and discuss relevant limitations

Given the limited sample sizes, RCTs included in the meta-analysis were not powered to detect an increased risk of IS after RV vaccination. Limitations such as the power of RCTs with regards to safety endpoints, and the appropriate risk period to detect IS could have been discussed more appropriately. Furthermore, the authors criticized study designs for large population-based studies. However, these types of studies (i.e. cohort or self-controlled case series) are robust and accepted by regulatory bodies, such as the European Medicine Agency and the United States Food and Drug Administration,^7^ as methods for assessing the association between vaccines and outcome events. The main advantage of case-only designs is that it inherently controls for all non-time varying confounders.^8^

## 4. Safety conclusion for newer vaccines is based on inadequate evidence

The meta-analysis included many RCTs for the 2 widely used vaccines (11 for *Rotarix*, 10 for *RotaTeq*) versus only 4 for the newer vaccines (i.e. 2 for *Rotasiil*, 1 for *Rotavac* and 1 for the unlicensed vaccine, RV3BB). This is likely to introduce bias in the interpretation. Furthermore, the authors concluded positively on the favorable benefit/risk profile of the 3 newer RV vaccines. However, only large-scale use of these vaccines can justify this conclusion. RCTs conducted for the 3 newer vaccines were restricted to 1 or 2 countries/regions and hence the generalizability of the findings is not appropriate. To date, no evidence from post-marketing studies or from pharmacovigilance activities are available for these vaccines. As continuous post-marketing safety monitoring is required for a more robust vaccine assessment,^7^ the review provides an incomplete and inadequate positive safety conclusion for the newer vaccines. Moreover, it is not appropriate to conclude that the newer vaccines’ benefit/risk profile will be comparable to that of the 2 widely used RV vaccines based on the currently limited evidence.

## 5. The study generates miscommunication and potential risk to confidence in RV vaccination

Scientific publications discussing the safety profile of vaccines should provide reliable information and inform accordingly the scientific community and the public at large about the benefits and risks of vaccines. Key findings on the association of IS with RV vaccination were published 10 years ago^6^ and paved the way for vaccine use recommendations taking into consideration this risk.^2-4^ This well-accepted evidence was omitted from the discussion and interpretation of the meta-analysis’ results and should have been considered.

It is, therefore, our intention to re-emphasize that extensive and updated evidence from post-marketing surveillance indicates a slight increased risk of IS, mostly within 7 days of RV vaccination for both widely used vaccines, suggesting a class effect among RV vaccines.^6^ This safety concern has been carefully evaluated by the WHO Global Safety evaluation committee and the Centers for Disease Control and Prevention, with the conclusion that the benefit of the widely used RV vaccines largely outweigh this slight increased risk of IS.^5^ In the context of RV vaccination, it remains important to educate health-care practitioners of the rare occurrence of IS, and in addition, to make them aware that the consequences of IS can be better managed with parental counseling, early diagnosis and timely treatment.^2,3^ In fact, early vaccination may even help to reduce the risk of IS by avoiding an overlap with the peak period of natural IS onset.^9^

While we appreciate the authors’ efforts and interest in contributing with a meta-analysis to a current and relevant topic, real-world data should not be excluded when analyzing the benefit/risk balance of vaccination, especially when vaccine safety is discussed. We, therefore, consider it imperative to bring to the attention of the scientific community, as well as the public, that the paper may contain incomplete and inadequately interpreted information, leading to misinterpretation and therefore misleading conclusions.

## Trademark statement

*Rotarix* is a registered trademark owned by the GSK group of companies. *RotaTeq* is a registered trademark of Merck&Co, Inc. *Rotavac* is a registered trademark of Bharat Biotech. *Rotasiil* is a registered trademark of Serum Institute of India Ltd.
